# To re-examine the intersection of microglial activation and neuroinflammation in neurodegenerative diseases from the perspective of pyroptosis

**DOI:** 10.3389/fnagi.2023.1284214

**Published:** 2023-11-09

**Authors:** Yuan Li, Ying-Jie Li, Zhao-Qiong Zhu

**Affiliations:** ^1^Department of Anesthesiology, Affiliated Hospital of Zunyi Medical University, Zunyi, China; ^2^College of Anesthesiology, Zunyi Medical University, Zunyi, China; ^3^Department of General Surgery, Mianyang Hospital of Traditional Chinese Medicine, Mianyang, China

**Keywords:** pyroptosis, microglia, neuroinflammation, signaling pathways, neurodegenerative diseases

## Abstract

Neurodegenerative diseases (NDs), such as Alzheimer’s disease, Parkinson’s disease, Huntington’s disease, and motor neuron disease, are diseases characterized by neuronal damage and dysfunction. NDs are considered to be a multifactorial disease with diverse etiologies (immune, inflammatory, aging, genetic, etc.) and complex pathophysiological processes. Previous studies have found that neuroinflammation and typical microglial activation are important mechanisms of NDs, leading to neurological dysfunction and disease progression. Pyroptosis is a new mode involved in this process. As a form of programmed cell death, pyroptosis is characterized by the expansion of cells until the cell membrane bursts, resulting in the release of cell contents that activates a strong inflammatory response that promotes NDs by accelerating neuronal dysfunction and abnormal microglial activation. In this case, abnormally activated microglia release various pro-inflammatory factors, leading to the occurrence of neuroinflammation and exacerbating both microglial and neuronal pyroptosis, thus forming a vicious cycle. The recognition of the association between pyroptosis and microglia activation, as well as neuroinflammation, is of significant importance in understanding the pathogenesis of NDs and providing new targets and strategies for their prevention and treatment.

## 1. Introduction

Neurodegenerative diseases (NDs) are a group of diseases characterized by neuronal damage and functional impairment, which are usually chronic, progressive, and irreversible, causing serious impact on patients’ quality of life and public health (Przedborski et al., 2003). With the continuous aging of the global population, the incidence and prevalence of NDs are increasing, so it is urgent to study effective prevention and treatment strategies. Recent studies have found that neuroinflammation and microglia play crucial roles in the development and progression of NDs (de Sousa et al., 2023). Microglia can polarize into M1 and M2 phenotypes, which exert pro-inflammatory and anti-inflammatory effects, respectively in neuroinflammation, thereby exerting protective or toxic effects on neuronal cells (Orihuela et al., 2016). At the same time, pyroptosis, also known as a type of programmed necrotic cell death, is one of the important pathways for neuronal death in NDs and is a strictly regulated cell suicide process. This process involves multiple pathological mechanisms such as inflammatory response, energy metabolism imbalance, oxidative stress, and protein aggregation, and microglia play a key role in these processes (Bussian et al., 2018). In this review, we first introduce pyroptosis, microglia, neuroinflammation, NDs and their relationship. Secondly, we discussed the non-insignificant role of multiple signaling pathways in the abnormal activation of microglia in chronic neuroinflammatory NDs exacerbating neuronal pyroptosis, providing new ideas for identifying potential prevention and therapeutic targets for NDs.

## 2. Pyroptosis

### 2.1. Pyroptosis as a phenotype of inflammatory cell death

Cell death can be divided into accidental cell death and regulated cell death (RCD). RCD involves signaling cascades mediated by effector molecules and is evolutionarily conserved, playing important roles in cellular biology and homeostasis maintenance. RCD occurring under physiological conditions is also known as programmed cell death, such as pyroptosis (Giacomini et al., 2023; Yu et al., 2023).

Pyroptosis is an extensively studied form of cell death that is distinct from apoptosis and necrosis (Wang R. et al., 2020). It has been observed in various diseases, including inflammatory diseases, neurological disorders, and cancer. It is characterized by nuclear DNA fragmentation, mitochondrial dysfunction, and cytoplasmic inflammation (Liu L. P. et al., 2021). Recent studies have indicated that pyroptosis is closely associated with inflammatory responses and serves as a phenotype of inflammatory cell death (Li et al., 2023). Gasdermin D (GSDMD) and mixed lineage kinase domain-like protein are key molecules mediating cell membrane destruction and the release of cell contents during pyroptosis (Kayagaki et al., 2015; Zhang et al., 2016). As a phenotype of inflammatory cell death, pyroptosis plays an important role in the inflammatory process. Inflammation is a protective response of the body to external stimuli. However, when inflammation is excessive or prolonged, it can lead to tissue damage and the development of diseases.

Pyroptosis can interact with the inflammatory response through multiple pathways. On one hand, the inflammatory response can activate signaling pathways of pyroptosis. For example, in the inflammatory process, mitochondrial dysfunction and reactive oxygen species (ROS) generation induced by damage signals and inflammatory factors can trigger pyroptosis (Lu et al., 2022). On the other hand, pyroptosis can also promote the occurrence and maintenance of inflammation. Studies have found that the intracellular molecules released by pyroptosis can act as messengers of inflammatory signals, further activating immune cells and enhancing the inflammatory response (Rogers et al., 2017).

### 2.2. Mechanisms and regulation of pyroptosis

Inflammasomes are multiprotein complexes that become activated in response to microbial and non-microbial proinflammatory triggers, and are assembled by pattern recognition receptors. It has been discovered that activated inflammasomes can activate members of the caspase family, such as caspase-1 and caspase-11, thereby activating the associated signaling pathways of pyroptosis (Ding and Shao, 2017). Some inflammasomes, such as NOD-like receptor family pyrin domain-containing 3 (NLRP3) inflammasome, can cleave and activate proinflammatory cytokines, such as interleukin-1β (IL-1β) and interleukin-18 (IL-18), through the mediation of caspase-1, leading to intensified inflammatory responses (Yang et al., 2019).

GSDMD is a membrane-binding protein with an N-terminal pore-forming domain and a C-terminal inhibitory domain (Liu Z. et al., 2019). Activated inflammasomes can cleave GSDMD into two fragments: an N-terminal fragment and a C-terminal fragment, through the action of caspase-1 or caspase-11. The N-terminal fragment can form pores on the cell membrane, allowing exchange of substances between the intracellular and extracellular environments, leading to cell dissolution and death (Zhang Y. et al., 2021). Activation of GSDMD by caspase-1 can cause cell membrane rupture, allowing the entry of molecules such as water into the cell, resulting in the release of large amounts of inflammatory cytokines, triggering inflammatory responses and pyroptosis (Hou et al., 2020). Recent studies have found that streptococcal pyrogenic exotoxin B cleaves GSDMA, inducing pyroptosis (Deng et al., 2022). Currently, the upstream and downstream signaling regulation mechanisms of the execution protein GSDMD in pyroptosis remain unclear.

Gasdermin E (GSDME) is another member of the GSDMD family, and its cleavage can also trigger pyroptosis (Wang Y. et al., 2017). Ectopic expression of mouse GSDME in BALB/c mouse 4T1E breast cancer cells significantly inhibited 4T1E tumor growth and led to increased tumor-associated macrophage and natural killer (NK cell) infiltration (Zhang et al., 2020). Furthermore, phorbol 12-myristate 13-acetate and ionomycin stimulation lead to increased NK cells and CD8+ tumor infiltrating lymphocytes in tumors expressing granzyme Band perforin (Loveless et al., 2021). Further research has shown that human NK cell line YT can induce pyroptosis in HeLa cells expressing GSDME. This induction is mediated by granzyme B, which not only cleaves GSDME at the same site as caspase-3 but also indirectly activates caspase-3 (Loveless et al., 2021).

Inflammatory factors, cellular stress, and pathological stimuli can all trigger pyroptosis. During the process of pyroptosis, the formation of the GSDMD pore leads to the release of cytoplasmic and intracellular organelles into the extracellular space, resulting in the massive release of inflammatory cytokines, causing inflammation and cell death (Ratajczak et al., 2021). Intracellular ROS, calcium imbalance, and ATP depletion also play a role in the regulation of pyroptosis (Wan et al., 2019). In addition, non-coding RNA and epigenetic modifications have been found to be involved in the regulation of pyroptosis. For example, miR-125a-5p triggers ox-LDL-induced pyroptotic factors in human umbilical vein endothelial cells by directly targeting Tet methylcytosine dioxygenase 2, leading to abnormal DNA methylation levels and enhanced ROS generation, ultimately resulting in pyroptosis (Zhaolin et al., 2019). In ApoE/ mice with high-fat diet treatment, oral administration of melatonin in the stomach can reduce the expression of pyroptosis-related genes NLRP3, apoptosis-associated speck-like protein containing CARD (ASC), caspase-1, Nuclear factor-kappa B (NF-κB)/GSDMD, and GSDMD n-terminal, and significantly reduce atherosclerotic plaques in the aorta (Zhang et al., 2018).

The mechanisms of pyroptosis are also regulated by various intracellular signaling pathways. The mitochondrial pathway involves processes such as the opening of the mitochondrial outer membrane permeability transition pore and signal transduction between the endoplasmic reticulum and mitochondria. The endoplasmic reticulum stress pathway involves the accumulation of partially denatured proteins in the endoplasmic reticulum and the activation of sensors such as IRE1, ATF6, and PERK (Missiroli et al., 2020). The release of cytochrome c is a typical feature of pyroptosis, which activates the caspase signaling pathway through the binding of cytochrome c with apoptotic protease-activating factor-1 (Xu W. et al., 2021). In addition, signaling pathways such as the NLRP3 inflammasome and ubiquitination are also involved in the regulation of pyroptosis (Heneka et al., 2013; Shi et al., 2022).

It has been demonstrated that there is a complex relationship between pyroptosis and immune reactions induced by pathological stress, and the involvement of pyroptosis in neuroinflammation mediated by microglia in NDs will be discussed in detail later.

## 3. Microglia

### 3.1. Microglial phenotypes

Microglia belong to the mononuclear phagocyte system, a cell family that includes progenitors in the bone marrow, monocytes in the circulating blood, and tissue macrophages in every organ including the central nervous system (CNS; Arnold and Betsholtz, 2013). Surprisingly, microglia act as macrophage-like cells with residual hematopoietic potential throughout life.

As intrinsic immune cells in the central nervous system, microglia play a role in surveillance and clearance of damaged or injured tissue, and are crucial for maintaining the homeostasis of the neural environment (Wu et al., 2023). Whether microglia exert beneficial or detrimental effects depends on various factors such as the type of stimulus and the duration of the impact, as transient activation of microglia usually has neuroprotective effects in NDs, but chronic or prolonged responsiveness of these cells can induce activation and lead to neuronal damage (Porro et al., 2021). Activated microglia comprise several subtypes with different gene expression profiles, cell morphologies, behaviors, and functions, suggesting a high degree of plasticity in microglia. Surprisingly, microglia can continuously transition between these different phenotypic subtypes in response to neuronal pathologies.

**Figure 1:**
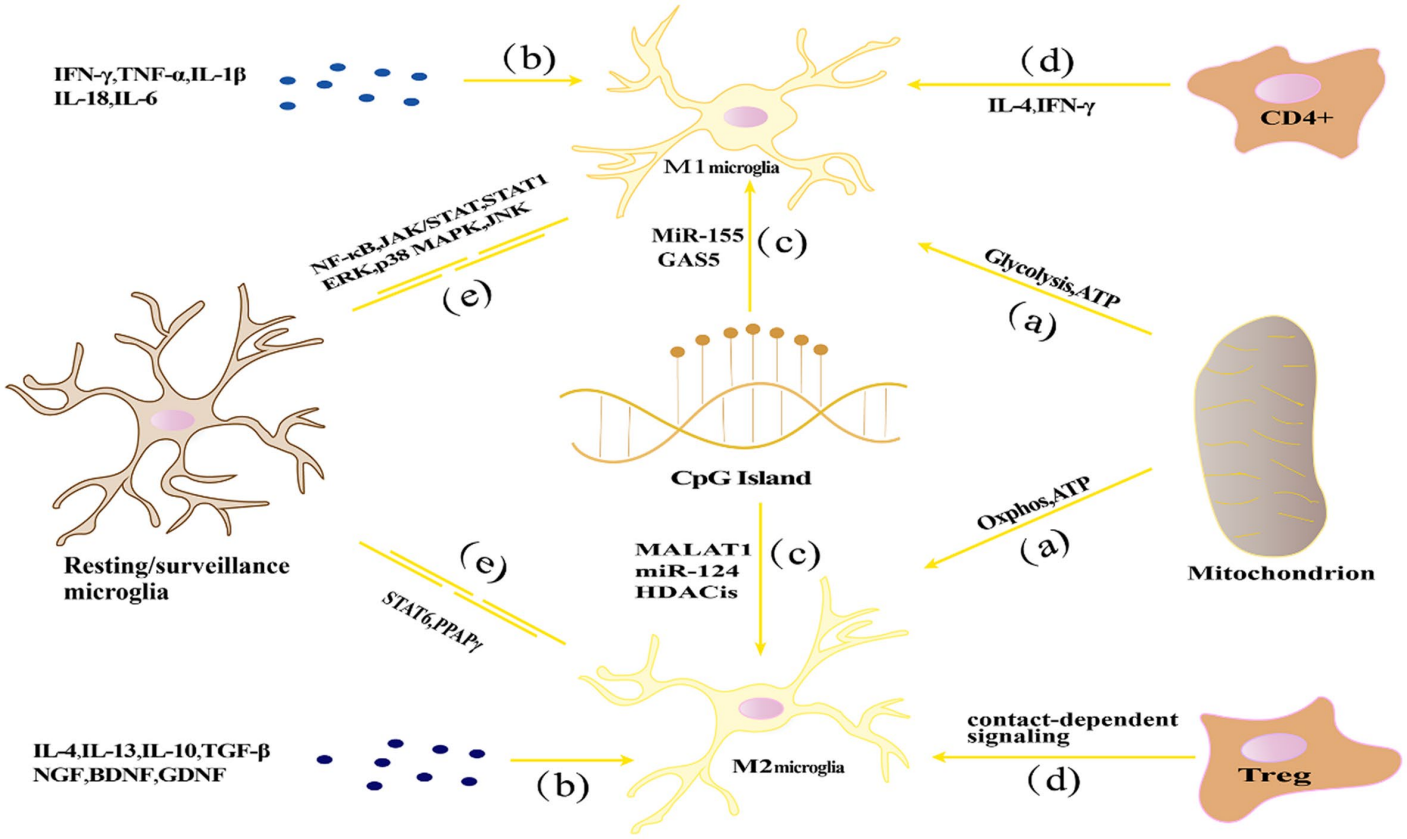
The activation and transformation of microglia between M1 and M2 subtypes. **(A)** Energy metabolism affects the polarization state through glycolysis and oxidative phosphorylation pathways, with M1 polarization relying primarily on glycolysis, while M2 polarization relies mainly on oxidative phosphorylation; **(B)** Cytokine networks influence microglial polarization by regulating processes such as inflammation and immune modulation; **(C)** Epigenetic regulation, such as DNA methylation, histone modifications, and non-coding RNA, can affect the expression of inflammation-related genes in microglia, thereby influencing their polarization state; **(D)** Microenvironmental factors such as cytokines, growth factors, chemical signaling molecules, and interactions with other cells can influence microglial polarization; **(E)** Signaling pathways including NF-κB, STAT, and MAPK pathways play important roles in regulating microglial polarization and immune regulatory functions.

Based on their activation environment or factors that stimulate them, microglia have been classified into “classically activated” M1 type (pro-inflammatory) and “alternatively activated” M2 type (anti-inflammatory), which are involved in the initiation and termination phases of inflammatory responses (Guruswamy and ElAli, 2017). Increasing evidence suggests that microglia could be a double-edged sword, exerting beneficial or harmful effects depending on the circumstances, which will be discussed in more detail later. Figure 1 represents the activation and transformation of microglia between M1 and M2 subtypes.

### 3.2. Mechanisms of microglial polarization regulation

The mechanisms regulating microglial polarization exhibit diversity and complexity. Firstly, energy metabolism plays a crucial role in microglial polarization, as different metabolic pathways lead to the differentiation of microglia into M1 (pro-inflammatory) or M2 (anti-inflammatory) polarized states. Secondly, the cytokine network significantly influences microglial polarization by precisely regulating processes such as inflammation and immune modulation. Additionally, epigenetic regulation is involved in microglial polarization by controlling gene expression patterns. Moreover, microenvironmental factors, such as neighboring cells, extracellular matrix, and local hormone levels, have important impacts on microglial polarization states. Finally, signaling pathways, including NF-κB, signal transducer and activator of transcription (STAT), and phosphoinositide 3-kinase/protein kinase B (PI3K/Akt), play critical roles in regulating microglial polarization. The following section will discuss in detail the homeostatic regulatory mechanisms of microglial polarization, including energy metabolism, cytokine network, epigenetic regulation, microenvironmental factors, and signaling pathways.

Energy metabolism and microglial polarization: Research has shown that energy metabolism regulation during microglia polarization is a key factor influencing its steady state (Bernier et al., 2020). Wang L. et al. (2023) suggested that mitochondrial dysfunction and oxidative phosphorylation disorder may cause microglial dysfunction and further affect neuronal survival. M1-type microglia mainly rely on glycolysis to produce energy, which can quickly generate ATP to meet the energy needs of M1-type microglia in inflammatory stress states. Studies have shown that glycolysis enhancement in M1-type microglia is closely related to the production of inflammatory mediators (Voloboueva et al., 2013). In addition, some signal pathways in M1-type microglia, such as the NF-κB and STAT1 signal pathways, also participate in the regulation of glycolysis-related enzymes, which affect cell energy metabolism (Meng et al., 2018). Conversely, M2-type microglia mainly rely on oxidative phosphorylation to produce energy. Enhanced mitochondrial function in M2-type microglia helps maintain a high level of oxidative phosphorylation, thus meeting its energy needs in neuronal repair and anti-inflammatory processes (Orihuela et al., 2016). Many M2 genes are regulated by transcription factors including STAT6 and PPARγ, which maintain mitochondrial oxidative phosphorylation (Hsu et al., 2023).

Cytokine network regulation: Cytokines play a crucial role in regulating neuronal injury and repair processes. Th1-type cytokines such as interferon-γ (IFN-γ) and tumor necrosis factor (TNF-α) as well as pro-inflammatory cytokines IL-1β, IL-18, interleukin-6 (IL-6) can induce microglia polarization toward the M1 type, while Th2-type cytokines such as IL-4 and interleukin-13 (IL-13) promote microglia polarization toward the M2 type (Perry et al., 2010; Cherry et al., 2014). IFN-γ and TNF-α promote the formation of M1-type microglia by activating signaling transduction and the STAT1 and NF-κB pathways to induce microglia to produce a large number of inflammatory cytokines, cytokines, and chemokines (Hu et al., 2015). In contrast, IL-4 and IL-13 induce microglia to express M2-type markers and promote anti-inflammatory and neuroprotective effects by activating the STAT6 signaling pathway (Junttila, 2018). In addition, growth factors such as nerve growth factor (NGF) and brain-derived neurotrophic factor (BDNF) play an important role in neuroprotection and repair. They promote microglia polarization toward the M2 type by activating specific signaling pathways, thus providing nutritional support for neurons (Kumar et al., 2016).

Epigenetic regulation: Epigenetics refers to the regulation of genetic information without changing the genetic sequence, including DNA methylation, histone modification, and non-coding RNA. In recent years, studies have shown that epigenetic regulation plays an important role in neuroinflammation and NDs (Berson et al., 2018).DNA methylation is an important way of epigenetic regulation. In the process of microglia polarization, DNA methylation can regulate the expression of inflammation-related genes. For example, CpG island methylation has been found to play a key role in regulating microglia M1/M2 polarization (Bhattacharjee et al., 2016). Histone modification, such as acetylation and methylation, also plays an important role in microglia polarization and steady-state regulation. Studies have shown that histone deacetylase inhibitors can induce microglia polarization toward the M2 type, thereby inhibiting inflammation (Wang et al., 2015). In addition, histone methyltransferase inhibitors can also regulate microglia inflammatory responses and polarization (Arifuzzaman et al., 2017). Non-coding RNAs, such as microRNAs (miRNAs) and long non-coding RNAs (lncRNAs), also play important roles in the polarization and homeostasis of microglia. For example, miR-155 and miR-124 have been identified as key regulators of M1 and M2 microglial phenotypes, respectively (Ponomarev et al., 2011; Cardoso et al., 2016). lncRNAs are also involved in regulating microglial polarization. Studies have shown that lncRNA H19, MALAT1, Gm4419, and SNHG14 have been reported to stimulate the activation of microglia toward the M1 phenotype and thereby promote neuroinflammation, lncRNA GAS5 has an inhibitory effect on M2 polarization of microglia (Sun et al., 2017; Wang J. et al., 2017; Wen et al., 2017; Han et al., 2018; Zhou et al., 2018).

Microenvironmental factors: Multiple factors in the neural microenvironment, such as cytokines, growth factors, chemical signaling molecules, and other intercellular interactions, collectively regulate the function and behavior of microglia. In neuroinflammation and NDs, the influence of microenvironmental factors on microglial polarization and homeostasis is crucial (Alexaki, 2021). Cytokines and growth factors released by neurons and astrocytes can regulate microglial polarization. For example, research has shown that TGF-βreleased by neurons can promote M2 polarization of microglia through regulation of IL-10 production (Butovsky et al., 2006). Whereas interleukin-34 (IL-34) released by astrocytes can influence microglial proliferation and polarization (Mizuno et al., 2011). Furthermore, chemical signaling molecules in the local microenvironment can also affect microglial polarization. For instance, ATP can induce M1 microglial polarization and promote inflammation through activation of P2X7 receptors (Lin et al., 2022). Whereas lipid mediators such as prostaglandin E2 can induce M2 polarization of microglia by acting on its EP2 receptor, thereby inhibiting inflammation (Fu et al., 2022). In addition, interactions between microglia and other immune cells can also influence their polarization and homeostasis. For example, CD4+ T cells affect the polarization of microglia by releasing IL-4 and IFN-γ, IL-4 promotes the polarization of M2 microglia, and IFN-γ promotes the polarization of M1 microglia (Beers et al., 2008; Barcia et al., 2012). Whereas regulatory T cells (Tregs) can promote M2 polarization of microglia via cell contact-dependent mechanisms (Gao et al., 2022). Under neuropathological conditions, substances generated from neuronal death and injury, such as nuclear acids, nuclear proteins, and mitochondrial components, can also activate microglia as ligands of damage-associated molecular pattern receptors (DAMPs), thereby triggering inflammatory reactions (Song et al., 2021). Furthermore, studies have shown that local oxygen concentration can also influence microglial behavior. Under hypoxic conditions, microglia can enhance neuroinflammatory responses by producing inducible nitric oxide synthase and pro-inflammatory cytokines (Liu S. et al., 2016). Therefore, improving local oxygen supply may have potential value for inhibiting neuroinflammation.

Signaling pathways: Multiple signaling pathways have been demonstrated to be involved in the activation, polarization, and immune regulatory functions of microglia, including the NF-κB pathway, Janus kinase (JAK)/STAT pathway, and mitogen-activated protein kinase (MAPK) pathway, among others (Merighi et al., 2022).

Research indicates that activation of the NF-κB pathway can induce M1 polarization of microglia, thereby promoting inflammation. Conversely, inhibiting NF-κB pathway activity can promote M2 polarization of microglia, reducing inflammation (Zhao R. et al., 2019). The JAK/STAT signaling pathway also regulates microglial polarization. Under neuroinflammatory conditions, activation of the JAK/STAT pathway can promote M1 polarization of microglia. Whereas inhibiting JAK/STAT pathway activity can promote M2 polarization of microglia and inhibit inflammation (Li J. et al., 2021). The MAPK signaling pathway is also involved in regulating microglial polarization. The MAPK pathway includes three major branches: extracellular signal-regulated kinase (ERK), p38 MAPK, and Jun N-terminal kinase (JNK). Research has shown that inhibiting the ERK or p38 MAPK pathways can alleviate inflammation and reduce M1 polarization of microglia (Hou et al., 2003). Whereas inhibiting the JNK pathway can promote M2 polarization of microglia and alleviate neuroinflammation (Xu et al., 2020). Various signaling pathways target the polarized phenotype of microglia and play an important regulatory role in neuroinflammation. At the same time, the signaling pathway is also a key pathway for neuronal pyroptosis, which will be discussed in detail at the end of this article.

## 4. Microglia, neuroinflammation, and pyroptosis in NDs

NDs are a group of progressive disorders characterized by the gradual loss and death of neurons. Although different types of NDs have their unique pathological features, they are closely associated with common mechanisms such as neuroinflammation, oxidative stress, abnormal protein aggregation, and cell death (Dugger and Dickson, 2017). In recent years, there has been extensive research on the pathogenesis and treatment strategies of NDs. It has been found that the deposition of β-amyloid (Aβ) within neurons is closely related to the occurrence of Alzheimer’s disease (AD), while abnormal aggregation of alpha-synuclein (α-syn) is considered a key factor in Parkinson’s disease (Wang Z. et al., 2019). Furthermore, mitochondrial dysfunction and oxidative stress in neurons also play important roles in many NDs (Jiang H. et al., 2021). Meanwhile, neuroinflammation is also considered as an important factor in the progression of NDs. Protein aggregation is a common pathological phenomenon in NDs and can induce neuroinflammation, thereby exacerbating protein aggregation and neurodegeneration. In fact, inflammation can occur even earlier than protein aggregation (Zhang W. et al., 2023). Neuroinflammation refers to the inflammation response that occurs in the nervous tissue due to infection, injury, immune reaction, or other pathological processes. It involves local infiltration of inflammatory cells, release of immune cells and mediators, as well as the pathological process of neuronal dysfunction (Kim et al., 2016). In neuroinflammation, pathological damage leads to impairment of nervous tissue and triggers an inflammatory response. Microglia and infiltrating immune cells accumulated in the inflamed area and releasing inflammatory mediators, such as cytokines, chemokines, and ROS, further induce tissue inflammation response (Jin et al., 2013; Becker, 2016).

In neuroinflammation, microglia are over-activated and proliferate. Activated microglia release inflammatory mediators such as IL-1β, nitric oxide, and prostaglandins, which have toxic effects on neurons and other cells (Suzumura, 2017; Liu et al., 2018; Subedi et al., 2019). The synthesis and release of pro-inflammatory cytokines are common features of neuroinflammation and microglial activation. M1 microglia, through the release of inflammatory cytokines, may exacerbate neuroinflammation, leading to neuronal damage and pyroptosis (Lin et al., 2018). Furthermore, M1 microglia can further impair neurons by producing nitrites and oxidative stress substances, while M2 microglia have anti-inflammatory and neuroprotective effects, which aid in the repair and regeneration of neural tissue (Beller et al., 2018).

As a highly complex and tightly regulated process, neuroinflammation is associated with a variety of cellular (e.g., microglia) and biological events (e.g., pyroptosis). Microglia can recognize various inflammasomes that trigger pyroptosis through surface receptors such as GSDMD and NLRP3, leading to activation, secretion of pro-inflammatory factors participation in neuroinflammation (Anderson et al., 2021). These findings suggest that pyroptosis and microglia may play a significant role in the occurrence and development of NDs through their involvement in neuroinflammation. In the process of pyroptosis, the activation of inflammasomes leads to the activation of caspase-1. Activated caspase-1 further cleaves and activates pro-inflammatory factors, particularly the cytokines IL-1β and IL-18, which are converted into their active forms and released into the extracellular environment, triggering neuroinflammation (He et al., 2015). Released IL-1β and IL-18 can bind to their receptors and activate downstream signaling pathways, such as NF-κB and MAPK, leading to an exacerbation of the inflammatory response, including the production of inflammatory mediators, expression of cell adhesion molecules, and regulation of other inflammation-related genes (Dinarello, 2018). Although Caspase-4/−5/−11 cannot directly cleave pro-IL-1β/−18 to activate Caspase-1, they are able to enhance the function of Caspase-1. Additionally, the activation of these Caspases can promote K+ efflux, further activating the NLRP3 inflammasome, thereby amplifying the inflammatory response and promoting neuroinflammation (Coll et al., 2022). The activation of inflammasomes can also induce the release of inflammatory mediators and modulate the activity of other effector molecules, such as high mobility group box 1 (HMGB1) and neutrophil extracellular traps, which accelerate the cascade of inflammation and further exacerbating neuroinflammation (Chen et al., 2018). During pyroptosis, the insertion of the N-terminal domain of GSDMD into the cell membrane forms GSDMD pores, disrupting the integrity of the cell membrane and resulting in the leakage of intracellular substances. These leaked cellular contents can activate immune cells and trigger an inflammatory response, further modulating inflammatory cell death and immune responses (Chen et al., 2016; Sborgi et al., 2016). The formation of N-GSDMD pores not only promotes the early release of the pro-inflammatory cytokine IL-1, triggering neuroinflammation, but it also accelerates the death of microglia (Long et al., 2023). Additionally, one of the characteristics of GSDMD pores is their small inner diameter, which prevents the direct release of larger pro-inflammatory factors such as TNF-α, IL-6, and HMGB1. These factors are typically only detectable after cell lysis and death occur. It is precisely due to this characteristic that pyroptosis plays a crucial role in the propagation of neuroinflammation (Liu et al., 2021a). Cellular and extracellular signaling molecules released during pyroptosis, such as heat shock proteins, ATP, DNA fragments, can be captured by recognition receptors on microglia, initiating inflammatory responses through Toll-like receptors (TLR) and NLR receptor families, known as “danger signals,” thereby activating microglia and inducing neuroinflammatory responses (Chen and Nuñez, 2010; Sait et al., 2021).

In early NDs, the activation of microglia and infiltration of pro-inflammatory cells both play a beneficial role. However, persistent inflammation can lead to significant neuronal damage, further exacerbating neurodegeneration, in which both microglial and neuronal pyroptosis play important roles. When there is excessive or persistent immune stimulation, microglia may undergo aberrant activation, leading to microglial pyroptosis, which releases a series of toxic molecules and proteins that exacerbate the gradual loss or degeneration of neurons (Wu et al., 2022). At the same time, pro-inflammatory factors can also stimulate neuronal pyroptosis, resulting in increased osmotic pressure of the cell membrane, cellular swelling and edema, ultimately leading to neuronal injury and degeneration (Long et al., 2023).AD is a common neurodegenerative disease, and the deposition of Aβ within neurons is closely related to its pathogenesis. In the early stages of AD, microglia in the patient’s body are activated by Aβ and clear Aβ deposition in the interstitium through receptor-mediated phagocytosis and degradation, thereby inhibiting its accumulation to some extent (Wu et al., 2022). However, as Aβ accumulates, microglia continue to be activated and produce excessive pro-inflammatory cytokines, thereby exacerbating neuroinflammation in AD patients. At the same time, Aβ can induce the activation of NLRP3 inflammasomes, leading to microglia pyroptosis (Wang S. et al., 2019). Another study showed that inhibiting the activation of NLRP3 inflammasomes can alleviate Aβ-induced memory impairment, suggesting that microglial pyroptosis plays a critical role in the progression of AD (Dempsey et al., 2017). Parkinson’s disease (PD) is another neurodegenerative disease with different pathological characteristics from AD, and it is associated with abnormal aggregation of α-syn within neurons. One study showed that α-syn can induce the activation of NLRP3 inflammasomes in macrophages and exacerbate neuroinflammatory reactions through microglial pyroptosis (Codolo et al., 2013). In BE (2)-M17 human dopaminergic neuroblastoma cells, it was found that the activated caspase-1 from inflammasomes can cleave α-syn *in vitro* and produce aggregates that are neurotoxic to neurons (Wu et al., 2022). In PD rat models induced by lipopolysaccharide (LPS) and 6-hydroxydopamine, it was found that the NLRP3 inflammasome components were highly expressed in microglia, and this result was reversed by the caspase-1 inhibitor (Ac-YVAD-CMK; Voet et al., 2019). This suggests that microglial pyroptosis may be associated with the pathological cleavage of extracellular α-syn and the formation of Lewy bodies. In motor neuron diseases, studies have found that the expression level of the pyroptosis marker GSDMD is increased in spinal cord samples of amyotrophic lateral sclerosis (ALS) patients, indicating that microglial pyroptosis may play an important role in the pathogenesis of ALS (Van Schoor et al., 2022). Furthermore, inhibiting NLRP3 inflammasome activation can delay neurodegeneration and disease progression in ALS mice, further confirming the role of pyroptosis in ALS pathogenesis (Johann et al., 2015).Huntington’s disease (HD) is a neurodegenerative disorder characterized by impaired motor control and cognitive function, which is associated with the mutation of the huntingtin (Htt) protein. Studies have found that mutated Htt protein can induce NLRP3 inflammasome activation in microglia, leading to microglia pyroptosis and neuroinflammation (Huang et al., 2011; Chen et al., 2022). Multiple sclerosis (MS) is a progressive central nervous system demyelinating disease characterized by the presence of multiple demyelinating plaques, neurodegeneration, and axonal transection or loss in the white matter. In MS, the activation of microglia is closely associated with neuroinflammation, demyelination, and neuronal damage (Ransohoff et al., 2015). Studies have shown that by regulating the polarization state of microglia and inhibiting the activation of M1 microglia and the production of pro-inflammatory cytokines, neuroinflammation and neuronal pyroptosis can be reduced during the process of remyelination in the central nervous system (Miron et al., 2013; Long et al., 2023).

The activation of microglia, neuroinflammation, and pyroptosis in NDs are closely interconnected and inseparable, existing as mutually dependent conditions. There is a bidirectional causal relationship between pyroptosis and microglial activation, as well as neuroinflammation. Pyroptosis can directly or indirectly cause microglia activation and neuroinflammatory response. Conversely, activated microglia and the neuroinflammatory environment can influence the occurrence and progression of microglia and neuronal pyroptosis. The following discussion will focus on the regulation of microglial activation on neuronal pyroptosis and the regulation of microglial pyroptosis, further exploring new avenues for the treatment and prevention of neurodegenerative diseases.

## 5. Microglia-mediated neuronal pyroptosis in neuroinflammation

In recent years, increasing studies have reported the complex relationship between neuronal pyroptosis and microglia in neuroinflammation. The process of pyroptosis is an ordered process activated by specific signaling pathways and has unique biological characteristics and molecular regulatory mechanisms. Microglia have dual effects in pyroptosis, promoting or mitigating it by regulating different phenotypes (M1/M2). On the one hand, microglia induce neuronal pyroptosis by releasing pro-inflammatory cytokines, causing neuroinflammation and activating signaling pathways related to pyroptosis (Liu et al., 2021b; Long et al., 2023). On the other hand, microglial cells can also inhibit the activation of pyroptosis-related signaling pathways, reduce neuroinflammation levels, and protect neurons from damage by producing antioxidant molecules and neurotrophic factors (Wang Y. et al., 2022). Therefore, exploring the interplay and regulatory mechanisms between microglia polarization and neuronal pyroptosis, particularly inducing M2 activation, holds promise as a novel and effective strategy for the treatment of neuroinflammatory and NDs. These strategies can improve the neuronal microenvironment, alleviate neuroinflammation, delay disease progression, and enhance patients’ quality of life.

### 5.1. Classical activation of microglia leads to neuronal pyroptosis

**Figure 2:**
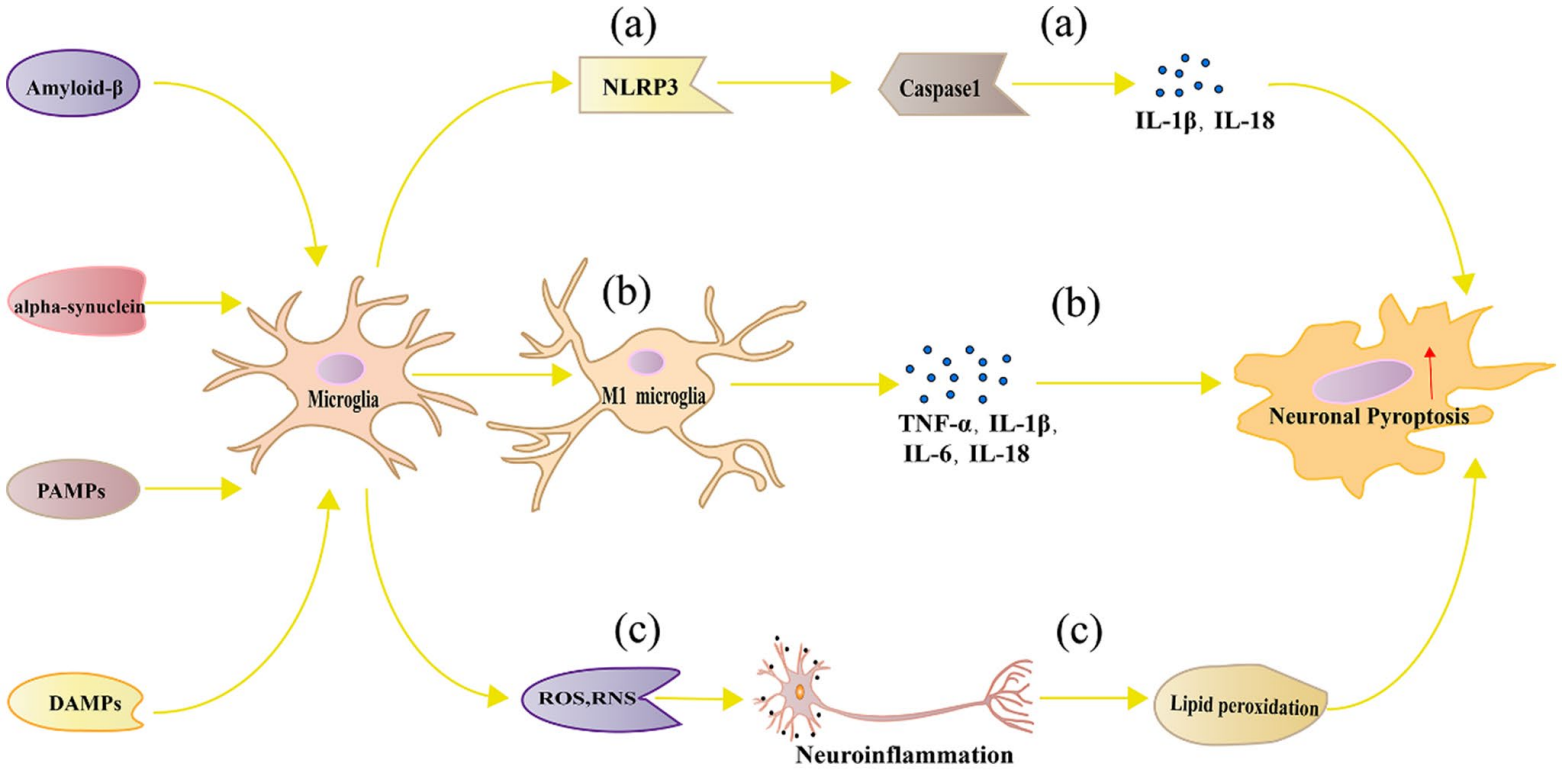
Mechanism of neuronal pyroptosis mediated by microglia. **(A)** Pathogen-associated molecular patterns, danger-associated molecular patterns, oxidative stress, and lipid metabolism dysregulation can activate the NLRP3 inflammasome in microglia, leading to neuronal pyroptosis through Caspase 1 activation; **(B)** Polarized M1 microglia release inflammatory cytokines such as TNF-α, IL-1β, IL-6, and IL-18, which can directly or indirectly cause neuronal damage and pyroptosis; **(C)** Microglia can generate ROS and RNS, which cause neuroinflammation and trigger neuronal pyroptosis.

Following classical activation, multiple pathways are simultaneously involved in microglia, triggering a series of cellular responses through various signaling pathways and molecular mechanisms, ultimately leading to neuronal pyroptosis. Below, we will provide a detailed description of the multiple pathways involved in neuronal pyroptosis induced by classical activation of microglia. Figure 2 represents the mechanism diagram of microglial classical activation-induced neuronal pyroptosis.

Activation of NLRP3 Inflammasome and Caspase-1 Activation: Under certain pathological conditions such as NDs and brain injuries, microglia can activate the NLRP3 inflammasome (Wu et al., 2021). The NLRP3 inflammasome is an intracellular multiprotein complex composed of NLRP3, ASC, and Caspase-1 (Martinon et al., 2009). Upon inflammatory stimulation, the NLRP3 inflammasome aggregates, resulting in the activation of Caspase-1 (Li et al., 2018). Activated Caspase-1 further cleaves and activates IL-1β and IL-18, inducing neuronal pyroptosis (Ding et al., 2022).

The activation of the NLRP3 inflammasome can be induced by various stimuli, such as pathogen-related molecular patterns (PAMPs), danger-associated molecular patterns (DAMPs), oxidative stress, and lipid metabolism disorders (Yang et al., 2019). Additionally, the activation of the NLRP3 inflammasome is also associated with mitochondrial damage and mitochondrial DNA release (Nakahira et al., 2011). In AD, the activation of the NLRP3 inflammasome is linked to Aβ deposition. Aβ can activate the NLRP3 inflammasome in microglia, leading to the release of IL-1β and IL-18, exacerbating neuronal damage and pyroptosis (Gustin et al., 2015; Hanslik and Ulland, 2020). Inhibiting the NLRP3 inflammasome can alleviate neuroinflammation and neuronal pyroptosis in AD mouse models (Hu et al., 2021b). PD is another neurodegenerative disease associated with NLRP3 inflammasome activation. Studies have found that the activation level of the NLRP3 inflammasome in microglia is significantly increased in PD patients and animal models (Lee et al., 2019). In PD, the aggregation of α-syn may induce the activation of the NLRP3 inflammasome in microglia, leading to neuronal damage and pyroptosis (Wu et al., 2022). Inhibiting the NLRP3 inflammasome can alleviate motor disorders and neuronal pyroptosis in PD mouse models (Wang W. et al., 2023). The activation of the NLRP3 inflammasome is also associated with MS and experimental autoimmune encephalomyelitis (EAE). In the EAE model, Ghrelin inhibition of NLRP3 inflammasome can significantly improve clinical symptoms and reduce neuronal pyroptosis (Liu F. et al., 2019). Furthermore, studies have found that the NLRP3 inflammasome is highly activated in the brain tissue of MS patients (Voet et al., 2018). These research findings suggest that the NLRP3 inflammasome may play a crucial role in promoting neuronal pyroptosis in microglia in NDs.

Release of Pro-inflammatory Cytokines: Pro-inflammatory cytokines released by M1-type microglia, such as TNF-α, IL-1β, and IL-6, can directly or indirectly induce neuronal damage and pyroptosis (Yang S. et al., 2021). TNF-α, an important pro-inflammatory cytokine, can initiate cell death signals on neurons by activating TNF receptor 1 (Feoktistova et al., 2021). TNF-α induces oxidative stress by inducing the production of endogenous ROS, leading to pyroptosis (Xi et al., 2020). Studies have shown that inhibiting TNF-α production or antagonizing its effects can alleviate neuronal damage and pyroptosis, improving the pathological changes in NDs (Capiralla et al., 2012). In animal models of NDs such as PD and AD, the inhibition of TNF-α can reduce neuronal pyroptosis and improve behavioral performance (Maurya et al., 2022). IL-1β is another key inflammatory cytokine that is closely associated with the activation of the NLRP3 inflammasome in microglia (Ye et al., 2019). IL-1β can induce neuronal inflammation and pyroptosis, leading to neurological damage (Savard et al., 2013). IL-1β can stimulate the activity of inducible nitric oxide synthase, leading to excessive production of nitric oxide, further triggering neuronal pyroptosis (Xu X. X. et al., 2023). Studies have shown that inhibiting IL-1β signaling has a protective effect in NDs such as AD and PD (Fulton et al., 2020). IL-6 has a complex role in neuroinflammation and neuroprotection. Additionally, IL-6 may promote pyroptosis by activating signaling transduction and transcriptional activation pathways (Capiralla et al., 2012). Increased expression of IL-6 has been observed in NDs including AD, PD, and MS (Lee et al., 2023). On one hand, IL-6 can promote inflammatory responses and exacerbate neuronal pyroptosis (Fink et al., 2008). On the other hand, IL-6 may also play a neuroprotective role under certain conditions (Behrens et al., 2008). IL-18 plays an important role in neuroinflammation and can influence neuronal survival by inducing IFN-γ production and modulating the activity of immune cells (Wawrocki et al., 2016). Elevated levels of IL-18 have been observed in NDs such as AD, PD, and MS. Inhibiting IL-18 activity can alleviate neuroinflammation and pyroptosis, and improve pathological presentations in animal models (Wang J. et al., 2022).

The actions of inflammatory cytokines in neuronal pyroptosis and NDs are interconnected. For example, TNF-α and IFN-γ can synergistically induce neuronal pyroptosis, and the interaction between IL-1β and IL-18 may further exacerbate pyroptosis responses (Chi et al., 2020; Karki et al., 2021). In addition, inflammatory cytokines can also affect the production of other cytokines such as chemokines and growth factors, thereby further regulating neuronal pyroptosis and repair processes (Borsini et al., 2015).

Oxidative stress and the generation of free radicals: Within neurons, an imbalance in redox reactions can lead to excessive production of ROS and reactive nitrogen species (RNS), further causing cellular damage and death (Hejna et al., 2021). Microglia can produce a large amount of ROS and RNS during the process of neuroinflammation, leading to pyroptosis (Park et al., 2015). On one hand, excessive ROS and RNS can directly damage cell membranes, proteins, and DNA, resulting in structural and functional impairments (Park et al., 2015). For example, ROS can induce lipid peroxidation, disrupting cell membrane integrity and fluidity, and eventually leading to neuronal pyroptosis (Yang et al., 2022). On the other hand, oxidative stress can regulate pyroptosis by activating signaling pathways. Studies have found that ROS and RNS can activate signaling pathways such as JNK and p38 MAPK, further inducing the expression of apoptotic proteins such as Bax and Caspase-3 (Wang Z. et al., 2018). Additionally, oxidative stress can promote neuroinflammation and neuronal pyroptosis by activating the NLRP3 inflammasome and NF-κB signaling pathways, leading to the production of inflammatory cytokines (Peng et al., 2020). Antioxidants such as N-acetylcysteine and alpha-lipoic acid can scavenge excessive ROS and RNS, reduce oxidative stress, and protect neurons from pyroptosis (Li et al., 2022). Furthermore, certain compounds derived from plants, such as flavonoids and resveratrol, possess antioxidant and anti-inflammatory properties, and can protect neurons from oxidative damage induced by microglia (Guo et al., 2016; Qi et al., 2020). In animal models of NDs, inhibiting ROS and RNS produced by microglia can improve neuronal function and reduce neuronal pyroptosis. For example, inhibitors of nitric oxide synthase such as 7-nitroindazole and 1,400 W can alleviate neuronal damage in PD animal models (Haik et al., 2008). Additionally, gene engineering techniques such as siRNA and gene editing can be used to regulate oxidative stress in microglia for the purpose of neuronal protection (Wang L. et al., 2018).

### 5.2. Replacement of activated microglia alleviates neuronal pyroptosis

**Figure 3:**
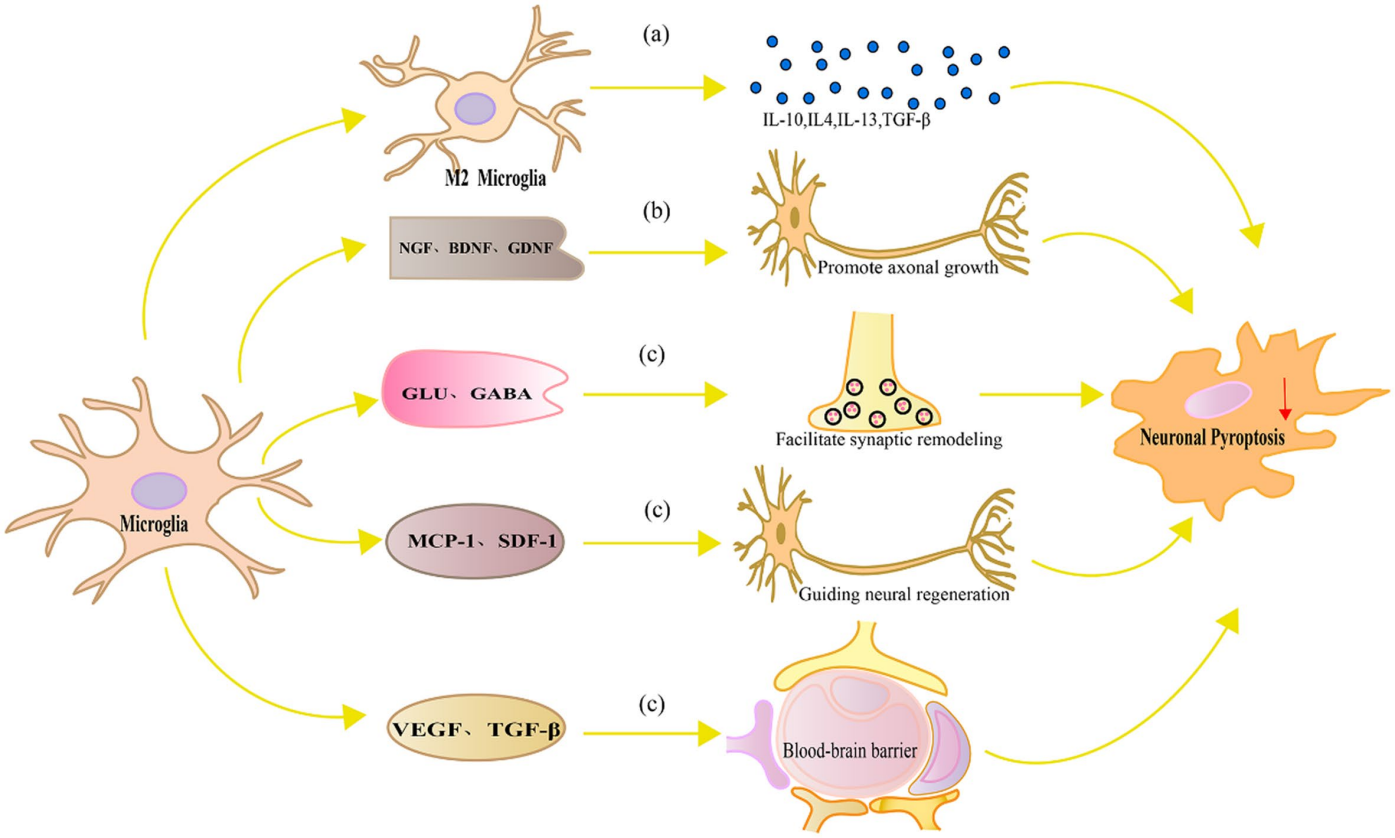
Mechanism diagram of replacement of activated microglia alleviates neuronal pyroptosis. **(A)** M2 microglia produce anti-inflammatory cytokines such as IL-10, IL-4, IL-13 and TGF-β, which can inhibit microglial activation, suppress the production of inflammatory cytokines, and consequently alleviate neuronal pyroptosis, reducing neuronal damage; **(B)** Neurotrophic factors such as NGF, BDNF, and GDNF promote axonal growth and regeneration in neurons, regulate signaling pathways, and enhance the ability of neurons to resist oxidative stress and pyroptosis; **(C)** Microglia can also facilitate synaptic remodeling and functional recovery by regulating local neurotransmitters and released modulatory factors, guiding neural regeneration, and maintaining the integrity and function of the blood–brain barrier, thereby alleviating neuronal damage and pyroptosis.

In contrast, replacement of activated microglia alleviates neuronal pyroptosis through multiple molecular mechanisms. Figure 3 represents the mechanism of replacement of activated microglia alleviates neuronal pyroptosis.

Production of anti-inflammatory factors: Microglia not only produce inflammatory cytokines but also produce anti-inflammatory cytokines, which protect neurons. In neuroinflammation and NDs, anti-inflammatory cytokines inhibit inflammation, regulate immune responses, and promote neuronal repair (Rajkovic et al., 2018).IL-10 is a typical anti-inflammatory cytokine that inhibits inflammation and immune responses (Crabtree et al., 2022). In neuronal injury and NDs, IL-10 can inhibit microglial activation, reduce the production of inflammatory cytokines, and thereby decrease neuronal pyroptosis and damage (Heinzerling et al., 2020). IL-10 can also regulate cytokine signaling pathways, such as inhibiting NF-κB and MAPK activities, further reducing the inflammatory response and neuronal pyroptosis (Yan et al., 2022). IL-4 and IL-13 are two anti-inflammatory cytokines with similar functions that mainly regulate microglial polarization, inducing microglia to transform into the M2 phenotype (Fu et al., 2022). M2 microglia have functions that inhibit inflammation, promote neuronal repair and growth (Wu et al., 2019). Studies have found that IL-4 and IL-13 can reduce neuronal pyroptosis and inflammatory responses and improve neuronal function in animal models of neuronal injury and NDs (Lee et al., 2013). In addition, IL-4 and IL-13 can reduce neuronal pyroptosis by inhibiting the production of inflammatory cytokines (Xu Y. et al., 2023). TGF-β is another anti-inflammatory cytokine that can regulate microglial activation and polarization and inhibit inflammation and immune responses (Herrera-Molina and von Bernhardi, 2005). In animal models of neuronal injury, TGF-β can reduce inflammatory damage, protect neurons from pyroptosis (Xu P. et al., 2021).

Release of neuroprotective factors: Neuroprotective factors are a type of bioactive protein that can protect neurons and support neuronal survival, growth, and differentiation. Microglia can release multiple neuroprotective factors under specific conditions to alleviate neuronal cell death. NGF is a growth factor with neuroprotective effects and can promote neuronal growth, differentiation, and survival. Studies have found that microglia can release NGF in the damaged nervous system environment, thereby protecting neurons from pyroptosis (Tang et al., 2022). In addition, NGF can regulate microglial activation and polarization, inducing microglia to transform into a phenotype with anti-inflammatory and neuroprotective functions (Rizzi et al., 2018). BDNF is another important neuroprotective factor that has protective, growth, and differentiation effects on neurons. Studies have shown that microglia can release BDNF, protecting neurons from inflammatory damage and oxidative stress-induced pyroptosis (Tang et al., 2022). In addition, BDNF can promote neuronal survival and resistance to cell death signals by regulating signaling pathways such as Akt and MAPK (Colucci-D'Amato et al., 2020). Glial cell line-derived neurotrophic factor (GDNF) is a growth factor with extensive neuroprotective effects, which can nourish various types of neurons, including dopaminergic neurons, motor neurons, parasympathetic and sympathetic neurons, as well as primary sensory neurons (Fundin et al., 1999). Under specific conditions, microglia can release GDNF to protect neurons from oxidative stress and inflammatory damage (Zhang et al., 2017). GDNF can activate ERK and PI3K signaling pathways by binding with its specific receptor GFRα1, promoting neuronal survival and antioxidant stress (Bogen et al., 2008). Microglia have important functions in nerve repair and regeneration in nerve injury and disease. By secreting growth factors, cytokines, and proteins that support intercellular interactions, microglia can promote neuronal functional recovery and nerve regeneration. Supporting axonal growth and regeneration is crucial for nerve repair and functional recovery. Studies have shown that microglia can secrete multiple axonal growth factors, such as NGF, BDNF, and GDNF, which can control the intrinsic growth ability of neurons and promote axonal growth and regeneration (Sandhu and Kulka, 2021).

Synapse is the key structure of information transmission between neurons, and synaptic remodeling and functional restoration are of great significance for the repair of nervous system. Microglia can promote synaptic remodeling and functional recovery by secreting cytokines such as BDNF, nerve activation factors, and neural adhesion molecules (Olude et al., 2022). Moreover, microglia can also regulate synaptic transmission and nerve function by regulating local neurotransmitters such as glutamate and γ-aminobutyric acid (Northrop and Yamamoto, 2011). Studies have shown that microglia can promote neural network function recovery by affecting synaptic homeostasis and plasticity in neuronal pyroptosis and NDs (Li et al., 2019). Nerve regeneration is a key process for restoring function after nerve injury. Microglia can secrete multiple growth factors such as vascular endothelial growth factor (VEGF) and fibroblast growth factor to promote proliferation, differentiation, and migration of endogenous neural stem cells (Rizvanov et al., 2011). Furthermore, microglia can also guide neurons and neural stem cells to migrate to the injury area through the release of chemotactic factors such as monocyte chemotactic protein-1 and matrix-derived growth factor-1, thereby promoting nerve regeneration (Qin et al., 2022).

Maintaining the integrity of the blood–brain barrier (BBB) is the key to maintaining the homeostasis of the nervous system. Microglia are involved in maintaining the integrity of the blood–brain barrier and reducing neuronal pyroptosis through multiple mechanisms. Their functional regulation helps protect the nervous system from injury and inflammation, and promotes neural repair and recovery. The disruption of the BBB after injury exacerbates neuronal pyroptosis (Li T. et al., 2021). Microglia can maintain the integrity and function of the blood–brain barrier through the secretion of cytokines such as TGF-β and VEGF (Wan et al., 2022). When the BBB is damaged, microglia respond quickly to participate in repair, remove harmful substances, maintain the balance of ions inside and outside neurons, and reduce neuronal pyroptosis (Michell-Robinson et al., 2015). Studies have shown that under conditions of nerve injury and inflammation, inhibition of TREM-1 can promote the polarization of microglia from M1 type to M2 type, reduce the destruction of the BBB, thereby alleviating neuronal damage and pyroptosis (Zhang et al., 2022).

Replacement of activated microglia play a crucial role in protecting neurons from pyroptosis. They achieve this by releasing anti-inflammatory cytokines, neuroprotective factors, and regulatory factors that facilitate neuronal repair and regeneration. This helps alleviate neuronal damage and improve NDs. Simultaneously, regulation of Microglia pyroptosis is also a potential approach for treating NDs. In the following, we will discuss specific strategies for regulation of Microglia pyroptosis.

### 5.3. Regulation of microglial pyroptosis for treatment of neurological diseases

For the treatment strategies targeting microglial pyroptosis, severally and widely studied targets include NLRP3, GSDMD and caspases.

Targeting NLRP3 has shown neuroprotective effects in inhibiting cell pyroptosis in NDs. In mouse microglia, inhibition of NLRP3 inflammasome using resveratrol can shift microglial phenotype from M1 to M2 and inhibit the NLRP3 inflammasome/pyroptosis axis, thereby reducing neuronal cell death (Ma et al., 2021). Curcumin improves cognitive impairment by inhibiting neuroinflammation-induced activation of microglia, modulating the TREM2/TLR4/NF-κB pathway, and reducing NLRP3-dependent pyroptosis (Zheng et al., 2021). Jiedu-Yizhi formula is a traditional Chinese medicine prescription formulated by renowned Chinese medicine master Ren Jixue based on the theory of “marrow deficiency and toxin damage” for treating AD. Jiedu-Yizhi formula reduces the expression of NLRP3, pro-caspase-1, and caspase-1 P20 proteins in the hippocampus of AD rats, inhibiting excessive activation of the NLRP3 inflammasome, suppressing GSDMD maturation, breaking the vicious cycle between Amyloid-beta and pyroptosis, and improving cognitive impairment in AD rats (Wang J. et al., 2022). MCC950 is a small molecule that inhibits the formation of the inflammasome by blocking NLRP3 activation, thereby inhibiting caspase-1 activation and microglial pyroptosis induced by LPS, reducing neurotoxic α-syn accumulation, dopaminergic neuronal damage, and improving behavioral deficits in a PD rat model (Wang R. et al., 2023). Glyburide, an oral antidiabetic medication, has been found to have the potential to treat NDs. It protects dopaminergic neurons in a mouse model of PD by inhibiting NLRP3 inflammasome activation, microglial M1 polarization, and oxidative stress (Qiu et al., 2021). Pre-treatment with quercetin, a flavonoid present in *Ginkgo biloba*, inhibits microglial activation through the NLRP3/IL-1β-dependent pathway, improving dopamine neuronal loss in LPS-induced PD mice (Han et al., 2021).

GSDMD is the ultimate downstream substrate in the pyroptosis pathway and closely related to cell death. Therefore, targeting GSDMD may be a feasible option for treating NDs. Necrosulfonamide, an early identified GSDMD inhibitor, can bind to GSDMD subunits, preventing their polymerization and cleavage, inhibiting the formation of p30 GSDMD pores, and reducing macrophage and microglial pyroptosis (Rathkey et al., 2018). Dimethyl fumarate, a potent antimicrobial preservative, has been FDA-approved for the treatment of MS despite its corrosiveness to the body. Recent research has found that dimethyl fumarate can prevent the interaction between GSDMD and caspase, limiting its processing and oligomerization and blocking microglial pyroptosis (Wang and Ruan, 2022). Paricalcitol, a vitamin D receptor activator, inhibits NLRP3, GSDMD, and caspase-1 expression, blocks NF-κB activation, and reduces pyroptosis. It is considered a potential candidate for treating NDs (Jiang S. et al., 2021). Baicalein, a bioactive flavonoid found in Scutellaria baicalensis, exerts its effects on PD treatment by inhibiting microglial pyroptosis through the NLRP3/GSDMD pathway, reducing dopaminergic neuronal loss, and suppressing pro-inflammatory cytokine increase (Wang J. et al., 2022). Salidroside, a natural compound found in certain plants, such as *Rhodiola rosea* and alpine rhododendron, has been found to reduce and inhibit microglial pyroptosis by lowering IL-1β and IL-18 expression, as well as reversing the elevated levels of TLR4, NF-κB, NLRP3, ASC, cleaved caspase-1, and cleaved GSDMD proteins in an AD mouse model (Cai et al., 2021). miRNA-22 attenuates microglial pyroptosis by targeting GSDMD, reducing NLRP3 inflammasome activation and inflammatory cytokine expression, and significantly improving memory and motor function in an APP/PS1 double-transgenic AD mouse model (Han et al., 2020).

VX-765, an orally available caspase-1 inhibitor, exhibits inhibitory effects on the HMGB1/TLR4/NF-κB inflammatory pathway and reduces microglial pyroptosis and polarization (Sun et al., 2020). VX-765 not only inhibits microglial pyroptosis induced by sevoflurane but also suppresses Aβ deposition and tau phosphorylation in microglia, suggesting its potential as a new therapeutic intervention for AD (Tian et al., 2021). Moreover, the use of VX-765 has been shown to increase the survival rate of neurons in PD cellular models and attenuate neural degeneration in transgenic MSA mice (Bassil et al., 2016; Wang et al., 2016). Belnacasan, an optimized version of VX-765, possesses higher specificity and stronger biological activity. Belnacasan blocks GSDMD cleavage and ASC oligomerization, reduces pyroptosis-related protein levels in the central nervous system, and alleviates EAE in mice (McKenzie et al., 2018). In HD, the activation of caspase-3 and other caspase members is associated with neuronal death and disease progression. Some studies have found that caspase inhibitors like Z-VAD-FMK can decrease caspase activity, reduce neuronal death, and improve HD pathology (Müller et al., 2011). Emricasan, an oral caspase inhibitor, selectively inhibits caspase-1, caspase-2, caspase-3, caspase-9, etc. It has been investigated as a candidate drug for the treatment of hepatitis, liver fibrosis, and liver cancer in several clinical trials, which showing potential in alleviating pyroptosis and inflammatory response (Frenette et al., 2019).

## 6. Multiple signaling pathways connecting microglia activation and neuronal pyroptosis

**Figure 4:**
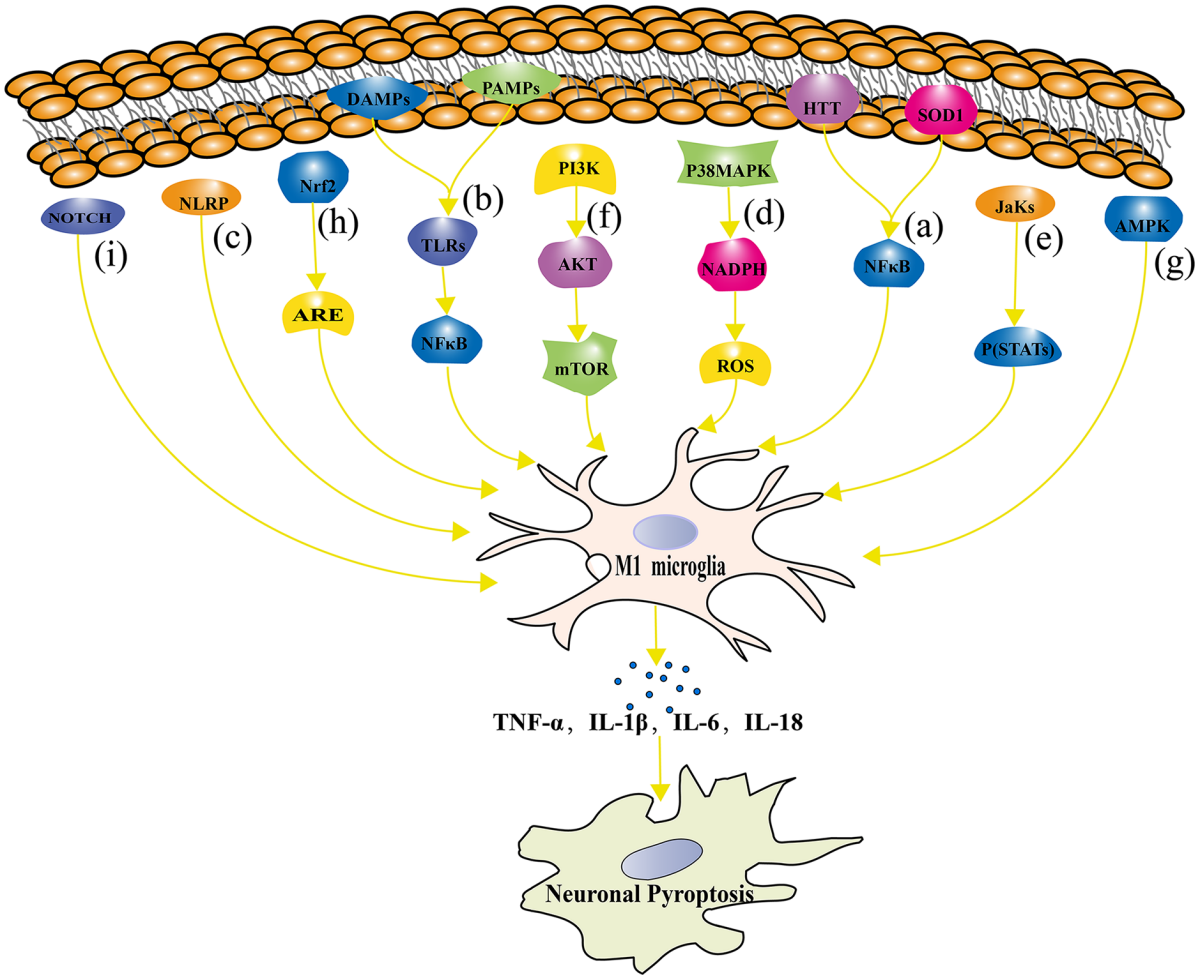
The signaling pathway connecting microglial polarization and neuronal pyroptosis. **(A)** The NF-κB signaling pathway regulates microglial polarization and plays a crucial role in neuronal pyroptosis; **(B)** TLRs signaling pathway is involved in the regulation of microglial polarization and its association with the development of NDs; **(C)** NLRP inflammasome directly contributes to neuronal pyroptosis; **(D)** The p38 MAPK signaling pathway regulates pyroptosis through oxidative stress damage; **(E)** The JAK–STAT signaling pathway plays an important role in microglial polarization and neuronal pyroptosis regulation in NDs such as AD, PD, and MS; **(F)** The mTOR signaling pathway inhibition leads to impaired neuronal growth, repair, and plasticity; **(G)** The AMPK signaling pathway reduces neuronal pyroptosis by inhibiting inflammatory responses and oxidative stress; **(H)** The Nrf2-ARE signaling pathway alleviates inflammation and neuronal pyroptosis by reducing oxidative stress; **(I)** The Notch signaling pathway influences microglial polarization and neuronal pyroptosis.

In order to understand the underlying mechanisms of microglial activation in neuronal pyroptosis, researchers have started to focus on the signaling pathways involved in microglial polarization and neuronal pyroptosis. Studies have shown that microglia can induce neuronal pyroptosis by activating the RIPK1 and RIPK3 signaling pathway through the release of TNF-α (Dhuriya and Sharma, 2018). In addition, microglia can regulate the process of neuronal pyroptosis through various signaling pathways, including TLRs, NLRP3 inflammasomes, p38 MAPK, JAK–STAT, PI3K/Akt/mTOR (mammalian target of rapamycin), AMPK, and nuclear factor erythroid 2-related factor 2 - Antioxidant Response Element (Nrf2-ARE). Figure 4 represents the signaling pathway connecting microglial polarization and neuronal pyroptosis.

### 6.1. NF-κB signaling pathway

NF-κB is a widely expressed transcription factor that is involved in the regulation of various biological processes, including immune response, inflammation, and cell survival (George et al., 2021). In NDs, aberrant activation of the NF-κB signaling pathway is closely related to neuronal pyroptosis and microglial polarization. On the one hand, the NF-κB signaling pathway plays an important regulatory role in microglial regulating M1 polarization of microglia. For example, in HD patients and mouse models, the activation of the NF-κB signaling pathway is closely related to microglial M1 polarization and the production of inflammatory cytokines (Mattson and Camandola, 2001; Simmons et al., 2016). On the other hand, inhibition of the NF-κB signaling pathway can promote microglial polarization toward M2, thereby exerting neuroprotective effects (He et al., 2021). In terms of pyroptosis, the activation of the NF-κB signaling pathway is closely related to neuronal damage. In PD, chronic activation of microglia releases inflammatory cytokines, leading to the activation of the NF-κB signaling pathway and aggravation of dopamine neuronal pyroptosis (Cai et al., 2022). Inhibition of the NF-κB signaling pathway can protect dopamine neurons from damage in PD models (Dou et al., 2018). Similarly, in HD research, mutated Htt protein can induce microglial activation, thereby activating the NF-κB signaling pathway and exacerbating neuronal pyroptosis (Björkqvist et al., 2008). Inhibition of the NF-κB signaling pathway can alleviate the inflammatory response of microglia and neuronal pyroptosis (Cianciulli et al., 2022). In ALS, the activation of the NF-κB signaling pathway is closely related to the inflammatory response and neuronal pyroptosis of microglia. It has been found that mutated superoxide dismutase 1 protein can induce microglial activation, thereby activating the NF-κB signaling pathway and exacerbating neuronal cell toxicity (Cardona et al., 2006). In addition, plasma exosomes pretreated with melatonin inhibited TLR4/NF-κB to reduce neuronal pyroptosis induced by cerebral ischemia, ultimately reducing ischemic infarct volume and promoting neurological recovery (Wang K. et al., 2020).

### 6.2. TLRs signaling pathway

TLRs are a class of membrane surface receptors present in immune cells such as microglia (Jack et al., 2005). TLRs play a crucial role in regulating immune responses, inflammation, and pyroptosis, especially in NDs. The TLRs signaling pathway plays an important role in the regulation of microglial polarization and neuronal pyroptosis. According to the study conducted by Li et al., TLRs in microglial activate specific downstream signaling pathways by recognizing PAMPs and DAMPs (Li Z. et al., 2021). This process further induces microglial M1 polarization, leading to the production of inflammatory cytokines such as TNF-α, IL-1β, and IL-6, and accelerating neuronal cell damage and pyroptosis (Arcuri et al., 2017). Further studies have shown that the key regulatory factors for TLRs signaling pathway activation are NF-κB and p38 MAPK (Akira et al., 2006). TLRs bind to their ligands and activate downstream signaling pathways through MyD88-dependent and MyD88-independent pathways. The MyD88-dependent pathway mainly activates the NF-κB signaling pathway, while the MyD88-independent pathway mainly activates the p38 MAPK signaling pathway, thereby promoting microglial M1 polarization and inflammatory response (Liu B. et al., 2021). In NDs, abnormal activation of the TLRs signaling pathway is closely related to the development and progression of the disease. For example, Kim et al. demonstrated in an animal model of PD that TLR2 acts as an α-syn receptor secreted by neurons, mediating the inflammatory response of microglia and leading to the loss of dopaminergic neurons (Kim et al., 2015). Similarly, the role of TLR4 in AD has also been widely studied. TLR4 is involved in Aβ-induced inflammatory responses and neuronal pyroptosis (Cai et al., 2021). Inhibition of the TLR4 signaling pathway can alleviate neuronal pyroptosis and cognitive impairment in AD models (Jin et al., 2019). In addition, TLR3 plays an important role in the CNS. TLR3 activation can induce microglia to produce inflammatory cytokines and chemokines, thereby exacerbating neuronal pyroptosis (Olson and Miller, 2004; Ramesh et al., 2013).

### 6.3. NLRP inflammasomes of NLR family pyrin domain

The inflammasome is a large protein complex that can sense multiple danger signals and activate the production of inflammatory peptides. The common signaling pathway of NLR family pyrin domain (NLRP) inflammasomes plays an important role in the polarization of microglia and neuronal pyroptosis. NLRP inflammasomes are a group of protein complexes responsible for regulating the inflammatory response process (Yang S. et al., 2021). Studies have found that the activation of NLRP inflammasomes is closely related to the occurrence and development of NDs (such as AD, PD, MS, etc.; Singhal et al., 2014). First, the activation of NLRP3 inflammasomes can promote the inflammatory response by inducing inflammatory cytokines (such as IL-1β, IL-18, etc.) produced by microglia (Scheiblich et al., 2017). Activated microglia release IL-1β, leading to neuroinflammation and further exacerbating neuronal pyroptosis (Swanson et al., 2019). In PD, Zhang et al. found that the activation of NLRP3 inflammasomes is related to microglia in dopaminergic neurons that are injured (Zhang C. et al., 2021). Secondly, the activation of NLRP inflammasomes can affect neuronal pyroptosis by regulating the polarization of microglia. For example, in PD, the activation of NLRP inflammasomes can cleave and activate pro-inflammatory cytokines, thus regulating the M1 and M2 polarization states of microglia and further affecting neuronal pyroptosis (Gustot et al., 2015). In addition, Li et al. revealed that Schisandrin improved cognitive impairment in AD mice through inhibition of NLRP1 inflammasome-mediated neuronal pyroptosis (Li Q. et al., 2021).

### 6.4. Highly activated p38 MAPK signaling pathway

The role of the p38 MAPK signaling pathway in the polarization of microglia and neuronal pyroptosis has received increasing attention. Studies have shown that the p38 MAPK signaling pathway plays a key role in microglial activation and inflammatory response, thus affecting neuronal survival and death (Morganti et al., 2019). Liu et al. found that after microglial activation, the activation of the p38 MAPK signaling pathway can induce the release of inflammatory cytokines, such as TNF-α, IL-1β, and IL-6. These inflammatory cytokines can further affect the survival and pyroptosis of neurons (Liu L. M. et al., 2016). In addition, Han et al. found that the p38 MAPK signaling pathway is involved in regulating the NADPH oxidase activity of microglia, thereby affecting the ROS levels produced by microglia and exacerbating oxidative stress damage and pyroptosis of neurons (Han and Choi, 2012). Zhang et al. found that the activation of the p38 MAPK signaling pathway in microglia is closely related to the expression of NADPH oxidase and macrophage Ag complex-1 during the process of dopaminergic neuronal damage induced by α-syn A30P and A53T mutations, further promoting oxidative stress damage and pyroptosis of neurons (Zhang et al., 2007).

### 6.5. JAK–STAT signaling pathway

The JAK–STAT signaling pathway is a typical cytokine signaling pathway that activates the JAK family of tyrosine kinases by binding to cytokine receptors, leading to the phosphorylation of STATs (Hu et al., 2021a). Phosphorylated STATs form dimers and are transferred to the nucleus to regulate the expression of target genes, affecting the polarization, proliferation, differentiation, and apoptosis of microglia (O'Shea and Plenge, 2012; Villarino et al., 2017). Studies have found that the JAK–STAT signaling pathway plays an important role in the polarization of microglia and neuronal pyroptosis regulation in NDs such as AD, PD, and MS. For example, in AD, it was found that inhibiting the JAK–STAT signaling pathway can reduce M1 polarization of microglia, thus alleviating neuronal pyroptosis and the inflammatory response (Yang L. et al., 2021). In PD research, the JAK–STAT signaling pathway is considered to be one of the key signaling pathways involved in the regulation of microglia activation, neuroinflammation, and dopaminergic neuronal pyroptosis (Fan et al., 2019). Moreover, in brain injury research, the JAK–STAT signaling pathway is considered to play a key role in microglial polarization. Researchers have found that inhibiting the JAK–STAT signaling pathway in a moderate traumatic brain injury mouse model can alleviate the inflammatory response and neuronal pyroptosis, indicating that it plays a critical role in disease development (Gao et al., 2020). In another study, treatment with JAK inhibitors ruxolitinib was found to reduce inflammation levels and alleviate pyroptosis (Zhao et al., 2021).

### 6.6. PI3K/Akt/mTOR signaling pathway

The PI3K/Akt/mTOR signaling pathway is the main pathway through which signals are transmitted via mTOR, and it plays an important role in mediating cell survival and proliferation (Savino et al., 2022). In the nervous system, the PI3K/Akt/mTOR signaling pathway is essential for neuronal growth, repair, plasticity, and protection. Aberrant activation of this pathway may lead to imbalanced polarization of microglia, which promotes neuronal pyroptosis and inflammatory reactions in the pathogenesis of NDs. Studies have found that activation of the PI3K/Akt/mTOR signaling pathway can counteract dopaminergic neuronal pyroptosis to alleviate the progression of Parkinson’s disease (Chen et al., 2019). Additionally, it has been demonstrated that rapamycin, an mTOR inhibitor, improves motor function and attenuates oxidative stress-related protein damage in a mouse model of Parkinson’s disease (Bai et al., 2015). In a rat model of spinal cord injury, Lu et al. found that activation of the PI3K/Akt/mTOR signaling pathway could regulate inflammation in spinal cord injury and promote axonal growth while reducing neuronal pyroptosis (Lu et al., 2023). In addition, Diao et al. revealed that hypothermia protects neurons against ischemia/reperfusion-induced pyroptosis via the PI3K/Akt signaling pathway (Diao et al., 2020). The PI3K/Akt signaling pathway activates both the NF-κB signaling pathway and its downstream pathways and promotes pyroptosis, as well as inhibiting autophagy mediated by PI3K/Akt/mTOR, while autophagy can inhibit the progress of pyroptosis (Shi et al., 2012; Deretic et al., 2013).

### 6.7. AMPK signaling pathway

AMPK is an important intracellular signaling molecule activated under conditions of energy metabolism disorder (Carling et al., 2012). The AMPK pathway plays a critical role in regulating neuroinflammation, oxidative stress, neuronal survival, and other processes and is therefore of great significance in the study of NDs. The regulatory role of the AMPK signaling pathway in microglial polarization and neuronal pyroptosis has been increasingly recognized. One study found that AMPK activation can promote M2-type microglial polarization, inhibit NLRP3 inflammasomes, and thus reduce neuronal pyroptosis (Tufekci et al., 2021). In another study, researchers found that AMPK activation had an inhibitory effect on M1-type microglial polarization, thus protecting neurons from damage (Wang C. et al., 2018). Researchers have found that bexarotene stimulates the AMPK-mTOR pathway in the cytoplasm and the AMPK-SKP2-CARM1 signaling pathway in the nucleus, promotes TFE3 translocation to the nucleus, activates autophagy and mitophagy, suppresses ROS production to alleviate oxidative stress, inhibits neuronal pyroptosis, and ultimately improves spinal cord rehabilitation after spinal cord injury (Wang X. et al., 2023). In addition, Zhao et al. revealed that amentoflavone could effectively reduce Abeta1-42-induced neurological dysfunction of an AD animal model via suppression of neuronal pyroptosis by targeting AMPK/ GSK3β signaling (Zhao N. et al., 2019).

### 6.8. Nrf2-ARE antioxidant signaling pathway

Nrf2 is a transcription factor that regulates the expression of a variety of antioxidant and detoxification genes by binding to AREs (Esteras et al., 2016). The Nrf2-ARE signaling pathway plays a key role in maintaining redox balance, regulating inflammatory responses, and resisting NDs. In recent years, many studies have found that the Nrf2-ARE signaling pathway plays a regulatory role in microglial polarization and neuronal pyroptosis. Nrf2 deficiency in a mouse model resulted in more severe neuronal injury and inflammatory reactions. In an animal model of traumatic brain injury, it was observed that Nrf2 deficient mice have enhanced NF-κB activation, inflammatory cytokine production in the brain compared to their wild type counterparts (Jin et al., 2008). Cheng et al. found that Nrf2, through the regulation of antioxidant enzyme expression, can reduce oxidative stress and thus alleviate inflammatory reactions and neuronal pyroptosis (Esteras et al., 2016). The activation of Nrf2 has been demonstrated to enhance antioxidant and anti-inflammatory properties, protecting neurons in models of neural injury (Dong et al., 2021). It has been found that Nrf2 activation can reduce neuronal pyroptosis by inhibiting inflammatory reactions, alleviating oxidative stress, and regulating energy metabolism (Cheng and Zhang, 2021).

### 6.9. Notch signaling pathway

The Notch signaling pathway is a key signal transduction pathway widely present in various cell types, regulating cell fate decisions, growth, and differentiation (Joosten et al., 2021). Recent studies have shown that the Notch signaling pathway plays an important role in the pathogenesis of NDs, especially in microglial polarization and pyroptosis. The activation of the Notch signaling pathway can affect the polarization state of microglia. It has been found that the inhibition of the Notch signaling pathway can promote the formation of M2-type microglia, which is beneficial for neuroprotection (Wu et al., 2019). Conversely, when the Notch signaling pathway is activated, it can induce microglia to polarize toward M1 type, leading to neuroinflammation and neuronal damage (Zhang Y. H. et al., 2023).

There is a correlation between Notch signaling pathway and pyroptosis. One study found that the Notch signaling pathway can regulate pyroptosis in endothelial cells under hypoxic/reoxygenation conditions. It was observed that the activation of the Notch pathway exacerbated pyroptosis, while inhibiting the Notch pathway reduced hypoxia/reoxygenation-induced endothelial pyroptosis (Zhang M. et al., 2023). Another study found that inhibition of the Notch-activating enzyme, γ-secretase, protected against ischemic neuronal cell death by targeting an apoptotic protease, cleaved caspase-3, NF-κB, and the pro-death BH3-only protein (Arumugam et al., 2011).

Multiple signaling pathways have been confirmed to play a role in the microglial polarization and neuronal pyroptosis, such as TLRs, NLRP inflammasomes, p38 MAPK, JAK–STAT, and PI3K/Akt/mTOR pathways. However, there are still many unanswered questions regarding the specific roles of these signaling pathways in NDs. Therefore, future research needs to further elucidate the detailed mechanism of these signaling pathways between microglial polarization and neuronal pyroptosis and evaluate their potential in therapeutic strategies. In addition, researchers also need to focus on the interaction and regulation between signaling pathways in order to better understand their roles in NDs. This will help discover new regulatory mechanisms and provide a wider range of target options for treatment.

## 7. Conclusion and future directions

Pyroptosis，the activation of microglia and neuroinflammation are involved in a vicious cycle that mutually influences each other and leads to the progression of NDs. In NDs, microglia are often activated and release cytokines, chemokines, and activators such as TNF-α, IL-1β, and NO, triggering neuroinflammatory responses. Activated microglia can directly or indirectly induce microglia and neuronal pyroptosis, and the released cytokines and activators can also induce microglia and neuronal pyroptosis. Neuroinflammation caused by inflammatory factors and cell infiltration further exacerbates neuroinflammation, activates microglia, and triggers more microglia and neuronal pyroptosis. This vicious cycle intensifies the progression of NDs, leading to more neuronal damage and death. The molecules and cellular processes involved in this vicious cycle are complex and dynamic, and further in-depth research is needed to fully understand its mechanism. However, the existence of this vicious cycle implies that simultaneously interfering with microglial activation, pyroptosis, and neuroinflammation may be key to treating NDs. By disrupting this vicious cycle, disease progression can be slowed down and neuronal function can be protected.

Protective effects on neurons or alleviation of neuroinflammation can be achieved by regulating the activation state of microglia. However, there are still many research topics that need to be further explored, including the signaling transduction pathways, epigenetic regulatory mechanisms, and homeostatic regulation mechanisms between microglial activation and neuronal pyroptosis. In future microglia studies, it is recommended to strengthen the investigation of the pyroptosis mechanisms of microglia and delve into their roles and regulatory mechanisms in the development of NDs. Additionally, comprehensive analysis of the signaling pathways linking microglia to neuronal pyroptosis should be conducted, which will help provide more accurate and effective approaches and strategies for the prevention and treatment of NDs.

## Author contributions

YL: Writing – original draft. Y-JL: Validation, Investigation, Writing – review & editing. Z-QZ: Conceptualization, Writing – review & editing.
